# 3-(2-Thioxo-1,3-dithiol-4-ylsulfan­yl)­propane­nitrile

**DOI:** 10.1107/S1600536808031711

**Published:** 2008-10-04

**Authors:** Bang-Tun Zhao, Jing-Jing Ding, Gui-Rong Qu

**Affiliations:** a College of Chemistry and Chemical Engineering, Luoyang Normal University, Luoyang 471022, People’s Republic of China; b College of Chemistry and Environmental Science, Henan Normal University, Xinxiang 453002, People’s Republic of China

## Abstract

The title compound, C_6_H_5_NS_4_, consists of a planar 2-thioxo-1,3-dithiol-4-ylsulfanyl unit [maximum deviation from the ring plane = 0.0325 (2) Å], with a cyano­ethyl­sulfanyl substituent in the 4-position. In the crystal structure, weak inter­molecular C—H⋯S hydrogen bonds together with S⋯N inter­actions [3.260 (5) Å] form two-dimensional layers in the *bc* plane.

## Related literature

For background to the chemistry of dithiole-2-thio­nes and tetra­thia­fulvenes, see: Chen *et al.* (2005 ▶); Fabre (2004 ▶); Segura & Martin (2001 ▶). For the preparation of the title compound, see: Liu *et al.* (2002 ▶). For a related structure, see: Jia *et al.* (2001 ▶).
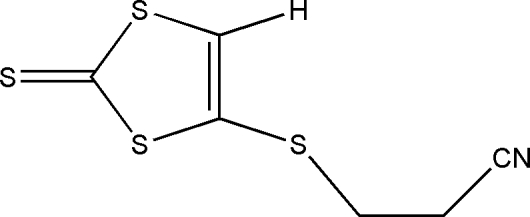


## Experimental

### 

#### Crystal data

C_6_H_5_NS_4_
*M* *_r_* = 219.35
Monoclinic, 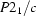

*a* = 5.2961 (9) Å
*b* = 10.8917 (19) Å
*c* = 16.031 (3) Å
β = 97.302 (2)°
*V* = 917.2 (3) Å^3^
*Z* = 4
Mo *K*α radiation
μ = 0.97 mm^−1^
*T* = 295 (2) K
0.35 × 0.27 × 0.23 mm

#### Data collection

Bruker SMART CCD area-detector diffractometer
Absorption correction: multi-scan (*SADABS*; Sheldrick, 1996 ▶) *T*_min_ = 0.728, *T*_max_ = 0.808
6626 measured reflections
1710 independent reflections
1525 reflections with *I* > 2σ(*I*)
*R*_int_ = 0.023

#### Refinement

*R*[*F* ^2^ > 2σ(*F* ^2^)] = 0.028
*wR*(*F* ^2^) = 0.072
*S* = 1.07
1710 reflections
100 parameters
H-atom parameters constrained
Δρ_max_ = 0.19 e Å^−3^
Δρ_min_ = −0.34 e Å^−3^

### 

Data collection: *SMART* (Bruker, 2000 ▶); cell refinement: *SAINT* (Bruker, 2000 ▶); data reduction: *SAINT*; program(s) used to solve structure: *SHELXS97* (Sheldrick, 2008 ▶); program(s) used to refine structure: *SHELXL97* (Sheldrick, 2008 ▶); molecular graphics: *SHELXTL* (Sheldrick, 2008 ▶); software used to prepare material for publication: *SHELXTL*.

## Supplementary Material

Crystal structure: contains datablocks I, global. DOI: 10.1107/S1600536808031711/sj2541sup1.cif

Structure factors: contains datablocks I. DOI: 10.1107/S1600536808031711/sj2541Isup2.hkl

Additional supplementary materials:  crystallographic information; 3D view; checkCIF report

## Figures and Tables

**Table 1:** Hydrogen-bond geometry (Å, °)

*D*—H⋯*A*	*D*—H	H⋯*A*	*D*⋯*A*	*D*—H⋯*A*
C5—H5*B*⋯S3^i^	0.97	2.86	3.813 (2)	167

Symmetry code: (i) 
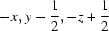
.

**Fig. 1.**
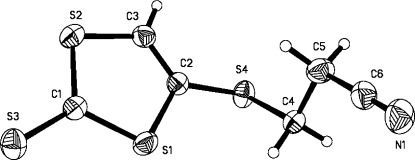
The molecular structure of the title compound with ellipsoids drawn at the 30% probability level.

**Fig. 2.**
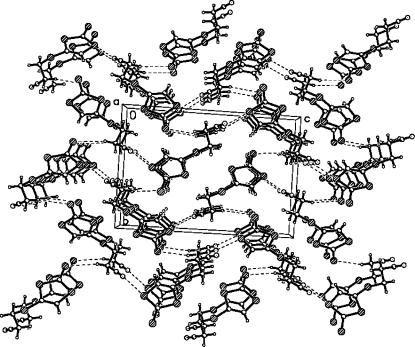
Crystal packing of the title compound viewed down the a axis.

## supplementary crystallographic information

### Comment

Tetrathiafulvalenes (TTFs) and their charge-transfer salts have become an interesting topic of research, due to their high electrical conductivity and superconducting properties (Segura & Martin, 2001). 1,3-Dithiole-2-thiones, important precursors to TTF derivatives, have also attracted attention (Chen,*et al.*; 2005; Fabre, 2004). In 2001, 4-alkylthio-1,3- dithiole-2-thione, a key kind of 1,3-dithiole-2-thiones was developed by a facile approach (Jia, *et al.*, 2001). We report here the structure of the title compound (Fig. 1), which was prepared by the reaction of di(tetraethylammonium) bis(1,3-dithiol-2- thione-4,5-dithiolate)zincate and 3-bromopropionitrile in the presence of pyridine hydrochloride.

The atoms of the five-membered dithiole ring and the doubly-bonded atom S3 are nearly coplanar, with a maximum deviation from the least-squares plane of only 0.0325 (2) Å (S1). However, S4 deviates considerably from the plane, 0.0775 (4) Å, which is very similar to the structure of 4,5-bis(2-cyanoethylthio)-1,3-dithiol-2-one (Liu,*et al.*, 2002). The cyanoethylsulfanyl group is substituted on the C2 atom of the dithiole ring. The C4—S4 bond length (1.8191 (19) Å) is typical of a single bond, while the other C—S bond lengths range from 1.650 (2) Å to 1.7435 (19) Å, suggesting a degree of conjugation in the dithiol-2-thione system.  In the crystal structure weak intermolecular C5—H5B···S3 hydrogen bonds, Table 1, together with S···N (3.260 (5) Å) interactions form two dimensional layers in the *bc* plane (Fig. 2).

### Experimental

The title compound was prepared according to the literature (Jia, *et al.*, 2001). Orange block-like single crystals were obtained from slow evaporation of a dichloromethane solution at room temperature.

### Refinement

All H-atoms were positioned geometrically and refined using a riding model with d(C-H) = 0.93Å,  U_iso_=1.2U_eq_ (C) for aromatic and 0.97Å,  U_iso_ = 1.2U_eq_ (C) for CH_2_ atoms.

### Crystal data

**Table:**

C_6_H_5_NS_4_	*F*(000) = 448
*M**_r_* = 219.35	*D*_x_ = 1.588 Mg m^−^^3^
Monoclinic, *P*2_1_/*c*	Mo *K*α radiation, λ = 0.71073 Å
Hall symbol: -P 2ybc	Cell parameters from 3529 reflections
*a* = 5.2961 (9) Å	θ = 2.6–27.9°
*b* = 10.8917 (19) Å	µ = 0.97 mm^−^^1^
*c* = 16.031 (3) Å	*T* = 295 K
β = 97.302 (2)°	Block, yellow
*V* = 917.2 (3) Å^3^	0.35 × 0.27 × 0.23 mm
*Z* = 4	

### Data collection

**Table:**

Bruker SMART CCD area-detector diffractometer	1710 independent reflections
Radiation source: fine-focus sealed tube	1525 reflections with *I* > 2σ(*I*)
graphite	*R*_int_ = 0.023
φ and ω scans	θ_max_ = 25.5°, θ_min_ = 2.6°
Absorption correction: multi-scan (SADABS; Sheldrick, 1996)	*h* = −6→6
*T*_min_ = 0.728, *T*_max_ = 0.808	*k* = −12→13
6626 measured reflections	*l* = −19→19

### Refinement

**Table:**

Refinement on *F*^2^	Primary atom site location: structure-invariant direct methods
Least-squares matrix: full	Secondary atom site location: difference Fourier map
*R*[*F*^2^ > 2σ(*F*^2^)] = 0.028	Hydrogen site location: inferred from neighbouring sites
*wR*(*F*^2^) = 0.072	H-atom parameters constrained
*S* = 1.07	*w* = 1/[σ^2^(*F*_o_^2^) + (0.0339*P*)^2^ + 0.3313*P*] where *P* = (*F*_o_^2^ + 2*F*_c_^2^)/3
1710 reflections	(Δ/σ)_max_ = 0.001
100 parameters	Δρ_max_ = 0.19 e Å^−^^3^
0 restraints	Δρ_min_ = −0.34 e Å^−^^3^

### Special details

**Table:**

Geometry. All e.s.d.'s (except the e.s.d. in the dihedral angle between two l.s. planes) are estimated using the full covariance matrix. The cell e.s.d.'s are taken into account individually in the estimation of e.s.d.'s in distances, angles and torsion angles; correlations between e.s.d.'s in cell parameters are only used when they are defined by crystal symmetry. An approximate (isotropic) treatment of cell e.s.d.'s is used for estimating e.s.d.'s involving l.s. planes.
Refinement. Refinement of *F*^2^ against ALL reflections. The weighted *R*-factor *wR* and goodness of fit *S* are based on *F*^2^, conventional *R*-factors *R* are based on *F*, with *F* set to zero for negative *F*^2^. The threshold expression of *F*^2^ > σ(*F*^2^) is used only for calculating *R*-factors(gt) *etc*. and is not relevant to the choice of reflections for refinement. *R*-factors based on *F*^2^ are statistically about twice as large as those based on *F*, and *R*- factors based on ALL data will be even larger.

### Fractional atomic coordinates and isotropic or equivalent isotropic displacement parameters (Å^2^)

**Table:**

	*x*	*y*	*z*	*U*_iso_*/*U*_eq_	
S1	0.21596 (10)	1.03249 (5)	0.12572 (3)	0.05315 (17)	
S2	0.34613 (11)	0.99269 (5)	0.30276 (3)	0.05650 (17)	
S3	−0.06717 (11)	1.17189 (6)	0.24268 (4)	0.05929 (17)	
S4	0.60268 (9)	0.85154 (5)	0.07595 (3)	0.05352 (16)	
N1	−0.1988 (4)	0.62697 (19)	−0.06631 (14)	0.0688 (5)	
C1	0.1519 (3)	1.07055 (18)	0.22542 (12)	0.0433 (4)	
C2	0.4470 (3)	0.92099 (17)	0.15433 (12)	0.0422 (4)	
C3	0.5061 (4)	0.90441 (19)	0.23696 (12)	0.0493 (5)	
H3	0.6287	0.8476	0.2583	0.059*	
C4	0.3347 (4)	0.79990 (19)	0.00199 (12)	0.0486 (5)	
H4A	0.2071	0.8642	−0.0051	0.058*	
H4B	0.3921	0.7852	−0.0522	0.058*	
C5	0.2159 (4)	0.6842 (2)	0.03091 (14)	0.0573 (5)	
H5A	0.3375	0.6174	0.0321	0.069*	
H5B	0.1748	0.6959	0.0876	0.069*	
C6	−0.0165 (4)	0.65095 (19)	−0.02465 (14)	0.0531 (5)	

### Atomic displacement parameters (Å^2^)

**Table:**

	*U*^11^	*U*^22^	*U*^33^	*U*^12^	*U*^13^	*U*^23^
S1	0.0531 (3)	0.0661 (3)	0.0389 (3)	0.0149 (2)	0.0007 (2)	0.0015 (2)
S2	0.0672 (4)	0.0630 (3)	0.0382 (3)	0.0049 (3)	0.0027 (2)	0.0019 (2)
S3	0.0548 (3)	0.0623 (4)	0.0621 (3)	0.0057 (2)	0.0123 (3)	−0.0081 (3)
S4	0.0401 (3)	0.0667 (3)	0.0525 (3)	0.0006 (2)	0.0008 (2)	−0.0142 (2)
N1	0.0643 (12)	0.0674 (12)	0.0708 (13)	−0.0096 (10)	−0.0057 (10)	−0.0105 (10)
C1	0.0420 (10)	0.0451 (10)	0.0424 (10)	−0.0085 (8)	0.0044 (8)	−0.0009 (8)
C2	0.0372 (9)	0.0432 (10)	0.0444 (10)	−0.0041 (7)	−0.0018 (7)	−0.0035 (8)
C3	0.0510 (11)	0.0464 (11)	0.0487 (11)	0.0034 (9)	−0.0009 (9)	0.0016 (9)
C4	0.0529 (11)	0.0505 (11)	0.0402 (10)	0.0001 (9)	−0.0022 (8)	−0.0024 (8)
C5	0.0569 (12)	0.0593 (13)	0.0531 (12)	−0.0052 (10)	−0.0030 (10)	0.0069 (10)
C6	0.0549 (12)	0.0503 (12)	0.0541 (12)	−0.0041 (9)	0.0070 (10)	−0.0044 (9)

### Geometric parameters (Å, °)

**Table:**

S1—C1	1.7262 (19)	C2—C3	1.334 (3)
S1—C2	1.7435 (19)	C3—H3	0.9300
S2—C3	1.727 (2)	C4—C5	1.508 (3)
S2—C1	1.729 (2)	C4—H4A	0.9700
S3—C1	1.650 (2)	C4—H4B	0.9700
S4—C2	1.759 (2)	C5—C6	1.470 (3)
S4—C4	1.8191 (19)	C5—H5A	0.9700
N1—C6	1.133 (3)	C5—H5B	0.9700
			
C1—S1—C2	97.91 (9)	C5—C4—H4A	109.1
C3—S2—C1	97.39 (9)	S4—C4—H4A	109.1
C2—S4—C4	101.59 (9)	C5—C4—H4B	109.1
S3—C1—S1	122.76 (12)	S4—C4—H4B	109.1
S3—C1—S2	125.08 (12)	H4A—C4—H4B	107.9
S1—C1—S2	112.15 (11)	C6—C5—C4	111.69 (18)
C3—C2—S1	115.08 (15)	C6—C5—H5A	109.3
C3—C2—S4	125.36 (15)	C4—C5—H5A	109.3
S1—C2—S4	119.25 (11)	C6—C5—H5B	109.3
C2—C3—S2	117.36 (16)	C4—C5—H5B	109.3
C2—C3—H3	121.3	H5A—C5—H5B	107.9
S2—C3—H3	121.3	N1—C6—C5	178.4 (2)
C5—C4—S4	112.31 (14)		
			
C2—S1—C1—S2	3.39 (12)	C4—S4—C2—S1	−52.73 (13)
C3—S2—C1—S3	178.04 (13)	S1—C2—C3—S2	0.7 (2)
C3—S2—C1—S1	−3.08 (12)	S4—C2—C3—S2	174.29 (11)
C1—S1—C2—C3	−2.54 (17)	C1—S2—C3—C2	1.47 (18)
C1—S1—C2—S4	−176.53 (11)	C2—S4—C4—C5	−78.01 (17)
C4—S4—C2—C3	133.94 (18)	S4—C4—C5—C6	173.85 (16)

### Hydrogen-bond geometry (Å, °)

**Table:**

*D*—H···*A*	*D*—H	H···*A*	*D*···*A*	*D*—H···*A*
C5—H5B···S3^i^	0.97	2.86	3.813 (2)	167

Symmetry codes: (i) −*x*, *y*−1/2, −*z*+1/2.
